# Circulating Angiogenic Cells can be Derived from Cryopreserved Peripheral Blood Mononuclear Cells

**DOI:** 10.1371/journal.pone.0048067

**Published:** 2012-10-25

**Authors:** Tanja Sofrenovic, Kimberly McEwan, Suzanne Crowe, Jenelle Marier, Robbie Davies, Erik J. Suuronen, Drew Kuraitis

**Affiliations:** 1 Division of Cardiac Surgery, University of Ottawa Heart Institute, Ottawa, Canada; 2 Department of Cellular and Molecular Medicine, University of Ottawa, Ottawa, Canada; 3 Department of Mechanical Engineering, University of Ottawa, Ottawa, Canada; 4 Department of Statistics, University of Ottawa Heart Institute, Ottawa, Canada; French Blood Institute, France

## Abstract

**Background:**

Cell transplantation for regenerative medicine has become an appealing therapeutic method; however, stem and progenitor cells are not always freshly available. Cryopreservation offers a way to freeze cells as they are generated, for storage and transport until required for therapy. This study was performed to assess the feasibility of cryopreserving peripheral blood mononuclear cells (PBMCs) for the subsequent *in vitro* generation of their derived therapeutic population, circulating angiogenic cells (CACs).

**Methods:**

PBMCs were isolated from healthy human donors. Freshly isolated cells were either analyzed immediately or cryopreserved in media containing 6% plasma serum and 5% dimethyl sulfoxide. PBMCs were thawed after being frozen for 1 (early thaw) or 28 (late thaw) days and analyzed, or cultured for 4 days to generate CACs. Analysis of the cells consisted of flow cytometry for viability and phenotype, as well as functional assays for their adhesion and migration potential, cytokine secretion, and *in vivo* angiogenic potential.

**Results:**

The viability of PBMCs and CACs as well as their adhesion and migration properties did not differ greatly after cryopreservation. Phenotypic changes did occur in PBMCs and to a lesser extent in CACs after freezing; however the potent CD34^+^VEGFR2^+^CD133^+^ population remained unaffected. The derived CACs, while exhibiting changes in inflammatory cytokine secretion, showed no changes in the secretion of important regenerative and chemotactic cytokines, nor in their ability to restore perfusion in ischemic muscle.

**Conclusion:**

Overall, it appears that changes do occur in cryopreserved PBMCs and their generated CACs; however, the CD34^+^VEGFR2^+^CD133^+^ progenitor population, the secretion of pro-vasculogenic factors, and the *in vivo* angiogenic potential of CACs remain unaffected by cryopreservation.

## Introduction

Although a unifying definition regarding their characterization does not exist [1], [2], endothelial progenitor cells (EPCs) often identified as CD34^+^VEGFR2^+^CD133^+^ cells have the ability to augment postnatal vasculogenesis [3]–[6]. The therapeutic revascularization that results from EPC transplantation is believed to be mediated by two main mechanisms: differentiation into new blood vessels [7], [8] and paracrine signaling to augment endogenous vessel growth via the production of pro-vasculogenic cytokines [9], [10]. In humans, patients with acute myocardial infarction who received an intracoronary infusion of bone marrow derived progenitors (sorted for markers CD34/CD45 and CD133) or peripheral blood-derived progenitors (plated for 3 days and positive for endothelial markers such as CD31, KDR (VEGFR2), von Willebrand factor and CD105 as well as uptake of low density lipoprotein and lectin binding) saw a beneficial effect in post-infarction remodeling processes, such as a global increase in ejection fraction and a decrease in infarct size [11], [12]. The number of EPCs in the blood has been shown to be a predictor of cardiovascular health: low levels of circulating EPCs have been associated with increased risk of major cardiovascular events and vascular function [13].

EPCs can be generated from the culture of peripheral blood mononuclear cells (PBMCs) isolated from blood by density gradient centrifugation. PBMCs are cultured for 4–7 days in endothelial-promoting media on fibronectin and the subsequently generated therapeutic population is referred to as ‘circulating angiogenic cells’ (CACs), or early EPCs [2], [14].

As cardiovascular disease is the number one leading cause of death in the Western world [15], there is a potential for CAC therapy to improve the quality of life for patients of this disease by aiding in the restoration of blood flow to the heart. However, EPCs and CACs are not available off-the-shelf and their frequency in circulating PBMCs is rather low, at about 0.0001% to 0.01% for EPCs [16] and 2% for CACs [17]. Furthermore, diabetes and cardiovascular disease decrease EPC numbers and function [18], [19], making it difficult to obtain therapeutically-relevant and potent cells for application in therapy. Cryopreservation offers a method to maintain cells as they are generated, until they are required for therapy. More importantly, cryopreservation may allow a patient to store his or her own autologous cells until needed, thereby avoiding the risks and potential of graft-versus-host disease [20].

Cryopreservation has been applied for some time in the medical field, ranging from freezing of blood and bone marrow cells for transplantation, to embryo preservation for *in vitro* fertilization and long term gamete storage for cancer patients. This process preserves cells by dramatically reducing biological metabolism at low temperatures; however, cryopreservation also causes damage to some cell types, as well as potentially changing their function [21], [22]. One study demonstrated that cryopreservation of T-cell subsets caused an increase in the expression of CXCR4 and CD69, while expression of L-selectin (CD62L) was decreased [23]. The consequence of cryopreservation on CACs, and their generation from PBMCs, remains to be thoroughly investigated.

The aims of this study were to investigate the outcome that cryopreservation has on the phenotype and function of: 1) freshly-isolated PBMCs; and 2) *in vitro* culture-generated CACs derived from fresh and cryopreserved PBMCs. In our study, we focused on the CACs (sometimes referred to elsewhere as early EPCs), which represent a highly heterogeneous population, thought to mostly exert their therapeutic effects through paracrine mechanisms.

## Results

**Figure 1:**
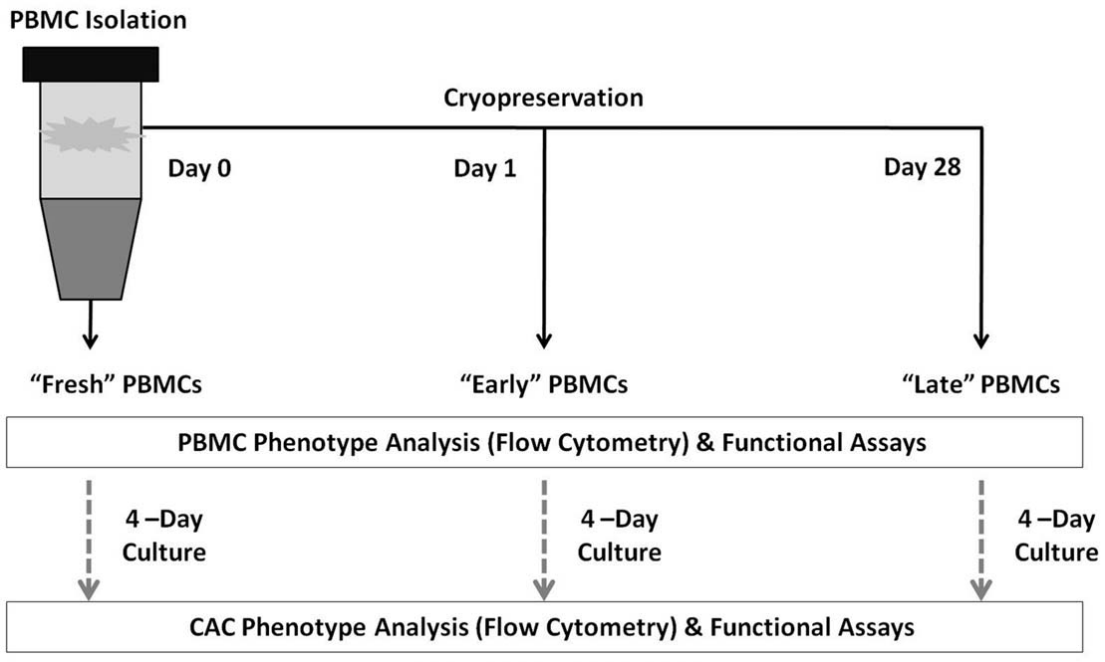
Summary of the methods. In brief, PBMCs were isolated and either analyzed (fresh), plated to generate CACs, or cryopreserved for 1 or 28 days after which the cells were thawed and analyzed or plated to generate CACs from cryopreserved PBMCs.

A summary of the cell populations and experiments is presented in Figure 1.

### Viability of PBMCs but not of Generated CACs is Reduced after Cryopreservation

**Figure 2:**
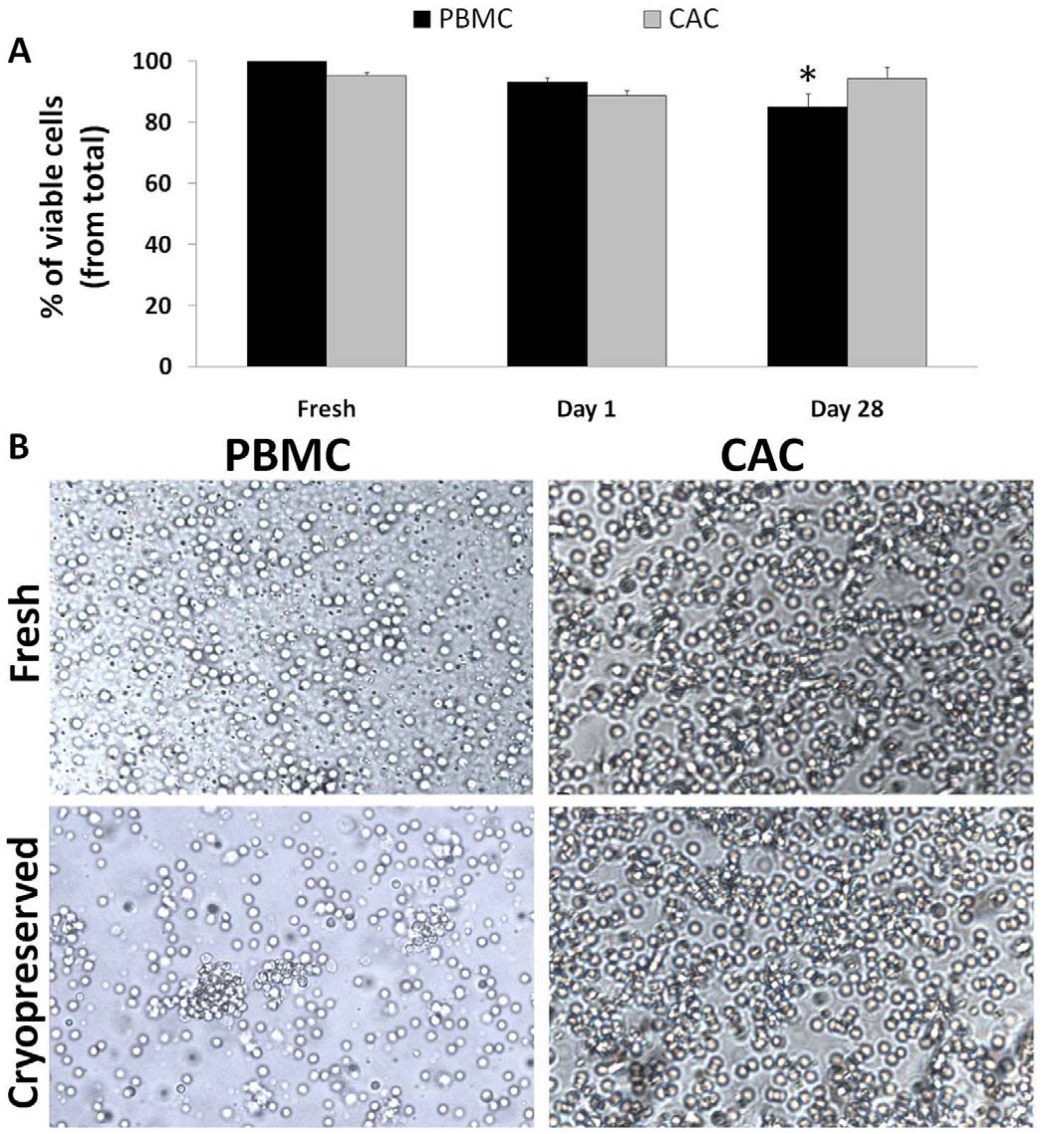
Viability of PBMCs, but not of generated CACs, is reduced after cryopreservation. 7AAD^–^ exclusion staining was used to enumerate the number of viable cells for fresh, early and late cryopreserved PBMCs and CACs. Prior to flow cytometry, the cells were incubated with 7AAD for 5 minutes. The proportion of viable cells is represented as a percentage ± SE (n = 7) (**A**). Representative images of fresh and cryopreserved PBMCs and CACs were taken by light microscopy at 10× magnification; scale bar = 50 µm. (**B**). *p<0.05 compared to the fresh sample.

Fresh and cryopreserved cells were incubated with 7AAD^-^ exclusion stain and the number of viable cells was quantified via flow cytometry. Fresh samples of PBMCs and CACs showed about 99.7±0.1% and 95.3±0.1% viability, respectively. Following cryopreservation, PBMCs sustained a non-significant reduction in viability on day 1 (93.1±1.5%) with a significant loss observed on day 28 (viability of 85.0±4.3%; *p* = 0.0078; Figure 2 A). However, the viability of CACs remained relatively stable over time post-cryopreservation at 88.7±1.4% on day 1 and 94.3±3.8% on day 28 (*p*_B_ = 1; Figure 2 A). Morphology of the thawed cells was preserved compared to their fresh counterpart sample as observed under a light microscope at 10× magnification (Figure 2 B).

### Cryopreservation Affects PBMC Phenotype

**Figure 3:**
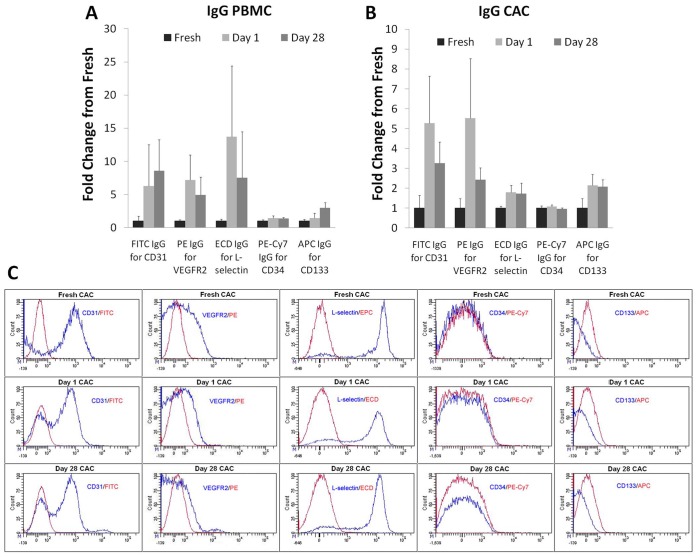
Isotype matched IgGs for each surface marker. Controls were conducted for background staining of fluorescent conjugates for markers CD31, VEGFR2, L-selectin, CD133 and CD34 on PBMCs (**A**) and CACs (**B**). No significant difference was observed in positively stained cells during fresh, day 1 and day 28 time points. The average IgG background staining is presented as fold-change **±** SE compared to fresh samples (n = 7). Representative histograms of the various markers (blue) and corresponding IgG (red) analysis from one donor’s fresh, day 1 and day 28 CACs (**C**).

The phenotype of the fresh and frozen cells was analyzed by staining the cells for surface markers: CD31, CD34, KDR (VEGFR2), CD133 and L-selectin and their appropriate isotype matched IgGs. The IgGs were used qualitatively [24] as there were no significant differences observed between the different sample time points (Figure 3 A–C). Surface markers CD34, KDR and CD133 were selected as they are most commonly used to describe a potent subset of CACs sometimes referred to as EPCs. Other surface markers investigated were CD31, an endothelial cell marker and L-selectin, an important adhesion protein for PBMCs and CACs. Expression of the endothelial marker CD31 remained stable at day 1 but by day 28 it was significantly increased compared to the earlier time points (Table 1). CD34 and VEGFR2 expressing cells followed a similar trend with a significant rise of these populations after cryopreservation compared to their fresh counterparts. Furthermore, the number of CD133 expression cells increased after cryopreservation while the number of L-selectin positive cells was reduced in the cryopreserved PBMC samples when compared to the fresh PBMC samples (Table 1). Double, triple and even quadruple staining of cells positive for the markers described above was also investigated to look at certain subpopulations of PBMCs. Their summary is shown in Table 1.

**Table 1:** PBMC phenotype is affected after cryopreservation.

PBMC	Fresh (%)	Day 1 (%)	Day 28 (%)
***CD31^+^***	67.38±2.17	70.42±4.12	76.88±2.10a,b
*CD31^−^CD34^+^*	10.12±0.47	7.69±0.62^a^	8.11±0.80a,b
*CD31^−^CD34^+^VEGFR2^+^*	0.61±0.14	1.55±0.17	2.16±0.15
*CD31^−^VEGFR2^+^*	1.64±0.39	3.88±0.42^a^	4.91±0.34^a^
*CD31^+^CD34^−^VEGFR2^+^*	3.36±0.57	8.04±0.73^a^	5.14±2.28^a^
*CD31^+^CD34^−^L-Selectin^+^VEGFR2^+^*	3.50±0.24	5.12±1.10	6.31±0.70^a^
*CD31^+^CD34^+^*	29.47±0.48	40.13±6.13	38.8±2.42
*CD31^+^CD34^+^VEGFR2^+^*	3.71±0.30	15.42±5.82	13.30±1.64
*CD31^+^L-Selectin^+^*	60.89±3.11	60.76±1.04	62.54±1.73
*CD31^+^VEGFR2^+^*	7.30±0.42	22.02±4.67	22.02±1.0
***CD34^+^***	35.89±1.36	48.14±2.72^a^	43.93±2.59^a^
*CD34^+^L-Selectin^−^*	4.72±0.82	5.42±0.89	4.72±1.02
*CD34^+^L-Selectin^-^VEGFR2^+^*	0.61±0.22	3.66±0.61^a^	2.58±0.76^a^
*CD34^+^L-Selectin^+^*	27.44±1.58	24.91±0.61	23.39±1.92^a^
*CD34^+^L-Selectin^+^VEGFR2^+^*	2.24±0.58	6.94±0.57^a^	4.63±2.15
*CD34^+^CD133^+^*	0.14±0.053	0.53±.077^a^	0.44±0.17
*CD34^+^CD133^+^VEGFR2^+^*	0.015±0.0082	0.15±0.043^a^	0.12±0.049
*CD34^+^VEGFR2^+^*	2.33±0.56	11.66±2.99^a^	7.63±2.81
***L-Selectin^+^***	84.37±1.52	74.79±2.43^a^	70.76±2.82^a^
*L-Selectin^−^VEGFR2^+^*	0.75±0.33	4.06±0.25^a^	3.57±0.61^a^
*L-Selectin^+^VEGFR2^+^*	3.66±0.56	6.40±0.79	5.08±1.61
***CD133^+^***	0.27±0.067	2.65±0.97^a^	1.97±0.23^a^
*CD133^+^VEGFR2^+^*	0.0027±0.0027	0.072±0.013^a^	0.084±0.031^a^
***VEGFR2^+^***	12.47±2.85	27.57±6.19^a^	27.07±5.44^a^

a *p*<0.05 vs. ‘fresh’ time point.

b *p*<0.05 vs. ‘day 1’ time point.

The values represent the percentage of cells within the total cell population (sample of 200,000 cells) that express the given phenotype.

### Cryopreservation Affects CAC Phenotype

The number of CACs generated was not significantly different between fresh and cryopreserved samples. CAC analysis followed the same procedures as the PBMCs and investigated the number of cells expressing the five markers: CD31, CD34, CD133, VEGFR2 and L-selectin. Overall, EPC identifying markers (CD34, VEGFR2 and CD133) did not show any significant differences after cryopreservation compared to the fresh samples (Table 2). The number of CD31 positive cells was significantly decreased after 1 day of cryopreservation; however, a significant decrease was not observed for the 28-day frozen cells. L-selectin was significantly up-regulated in cells after 28 days of cryopreservation compared to the fresh samples. The double, triple and quadruple staining of cells for the selected markers described was also investigated to look at various subpopulations of CACs. Their summary is shown in Table 2.

**Table 2:** CAC phenotype is affected after cryopreservation.

CAC	Fresh (%)	Day 1 (%)	Day 28 (%)
***CD31^+^***	72.81±2.94	61.97±1.09^a^	58.10±5.12
*CD31^−^CD34^+^*	8.38±1.25	12.51±0.58^a^	12.98±0.78^a^
*CD31^−^CD34^+^VEGFR2^+^*	0.79±0.23	2.70±0.32^a^	1.76±0.53
*CD31^−^VEGFR2^+^*	1.80±0.50	7.35±0.96^a^	4.62±1.20
*CD31^+^CD34^−^VEGFR2^+^*	3.83±0.66	8.83±0.32^a^	6.80±0.83^a^
*CD31^+^CD34^−^L-Selectin^+^VEGFR2^+^*	3.05±0.51	6.88±0.26^a^	4.65±0.72
*CD31^+^CD34^+^*	31.45±1.68	29.20±1.20	19.39±3.24^a^
*CD31^+^CD34^+^VEGFR2^+^*	3.81±0.63	8.42±0.89^a^	5.27±0.82^b^
*CD31^+^L-Selectin^+^*	64.26±2.69	58.45±1.36	45.79±6.69^a^
*CD31^+^VEGFR2^+^*	6.75±1.08	16.04±1.14^a^	11.19±1.63
***CD34^+^***	42.53±1.89	42.73±2.17	38.51±3.26
*CD34^+^L-Selectin^−^*	4.53±0.82	2.69±0.37	2.18±0.61
*CD34^+^L-Selectin^−^VEGFR2^+^*	0.90±0.19	1.95±0.29^a^	0.78±0.24^b^
*CD34^+^L-Selectin^+^*	31.29±1.61	28.03±1.06	23.78±3.27
*CD34^+^L-Selectin^+^VEGFR2^+^*	3.44±0.54	9.32±1.16^a^	6.31±1.29
*CD34^+^CD133^+^*	0.24±0.11	0.71±0.25	4.35±2.43
*CD34^+^CD133^+^VEGFR2^+^*	0.070±0.042	0.23±0.064	0.44±0.22
*CD34^+^VEGFR2^+^*	3.57±0.63	10.07±1.28	5.88±0.99
***L-Selectin^+^***	82.99±2.65	87.26±1.47	92.04±1.98^a^
*L-Selectin^−^VEGFR2^+^*	1.00±0.31	2.88±0.36^a^	2.83±0.72^a^
*L-Selectin^+^VEGFR2^+^*	3.97±0.67	10.34±0.67^a^	6.32±1.30^b^
***CD133^+^***	2.54±1.15	4.93±1.02	3.60±1.89
*CD133^+^VEGFR2^+^*	0.050±0.030	0.12±0.022	2.39±1.40
***VEGFR2^+^***	14.67±3.15	26.26±2.19	28.44±6.47

a *p*<0.05 vs. ‘fresh’ time point.

b *p*<0.05 vs. ‘day 1’ time point.

The values represent the percentage of cells within the total cell population (sample of 200,000 cells) that express the given phenotype.

### Cryopreservation does Affect Lectin Binding and LDL Uptake of CACs

**Figure 4:**
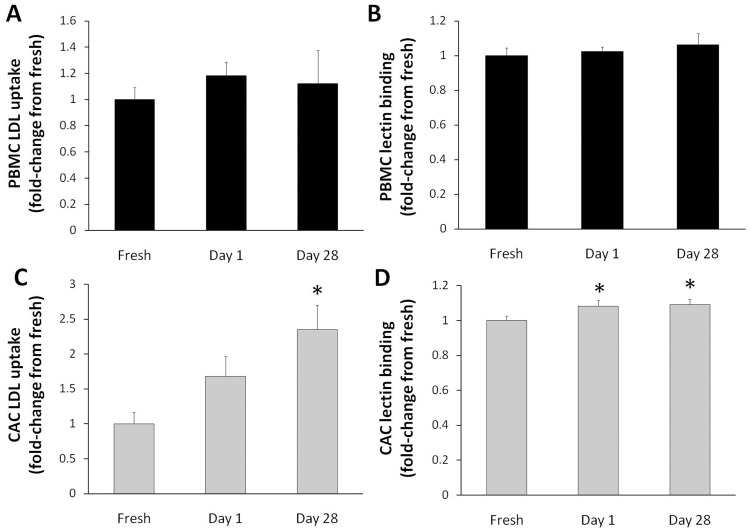
Cryopreservation affects LDL uptake and lectin binding of CACs. The numbers of positive PBMCs that took up fluorescent LDL (**A**) or that bound fluorescent lectin (**B**) were quantified using flow cytometry. The same procedure was conducted for CACs (**C**, **D**). Data is reported as fold-change from donor-matched fresh samples **±** SE (n = 7). *p<0.05 compared to the fresh sample.

Lectin binding and LDL uptake are characteristic functions of EPCs and other circulating cells, such as leukocytes. We decided to investigate whether these functions are altered in the cells by the cryopreservation process. There were no significant differences in the uptake of LDL and binding of lectin when day 1 and day 28 cryopreserved PBMCs were compared to the fresh PBMCs (Figure 4 A, B). However, a significant increase in LDL uptake (by 2.4-fold) was observed in day 28 CACs generated after cryopreservation, compared to fresh CACs (Figure 4 C, *p* = 0.004). There was also a significant increase in lectin binding for CACs after cryopreservation with an increase of ∼1.1-fold compared to the fresh samples (Figure 4 D, *p* = 0.005).

### Functional Capacities of PBMCs and CACs after Cryopreservation

**Figure 5:**
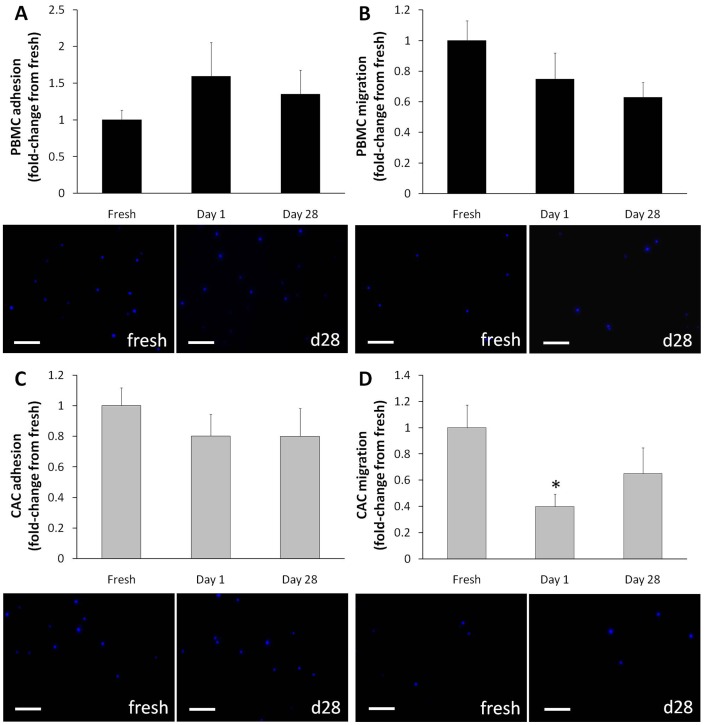
Cryopreservation does not affect adhesion and migration of PBMCs and CACs. PBMCs were pre-stained with DAPI for visualization by electron microscope for analysis of their adhesive (**A**) and migration (**B**) capabilities. The same procedure was performed for CACs (**C, D**). Five pictures were taken per well and the number of DAPI^+^ cells (blue) were counted per FOV and representative pictures of each assay are shown. Data is reported as fold-change from donor-matched fresh samples **±** SE (n = 7), scale bar = 80 µm. *p<0.05 compared to the fresh sample.

Adhesion and migration are important functional properties of most cells. Since changes in some of the proteins involved in these processes (VEGFR2, L-selectin) were observed after cryopreservation, assays measuring the cells` adhesive and migratory abilities were conducted. Overall, cryopreserved PBMCs and CACs demonstrated no difference in adhesive capabilities on fibronectin coated plates compared to the fresh samples (Figure 5 A, C). Cryopreserved PBMCs were also unaffected in their ability to migrate using a VEGF chemokine (Figure 5 B), whereas CACs after 1 day of cryopreservation exhibited reduced migration (*p* = 0.04), compared to fresh CACs (*p* = 0.001). Migration of day 28 cryopreserved CACs was not significantly affected (*p* = 0.12; Figure 5 D).

### Cytokine Secretion of CACs after Cryopreservation

**Figure 6:**
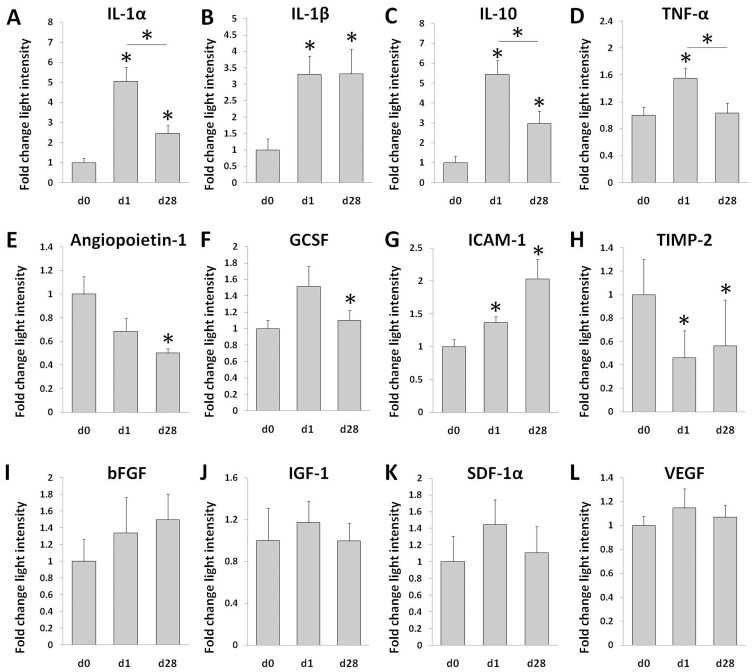
Cytokine secretion of CACs after cryopreservation. Fresh and cryopreserved PBMCs were cultured for 4 days to generate CACs. The media was collected and analyzed using a cytokine array (n = 5−6). Results of some of the cytokines are shown as a fold-change in light intensity ± SE; (**A–L**). *p<0.05 compared to the fresh sample, unless otherwise indicated.

The supernatant from CAC cultures was collected and a cytokine array was performed to evaluate the secretion of select cytokines by the cells. The secretion of pro-inflammatory cytokines interleukin-1α (IL-1α), IL-1β and tumor necrosis factor (TNF-α) and anti-inflammatory cytokine IL-10 was observed to be higher in frozen cells compared to their fresh counterparts. In most cases, day 1 cryopreserved cells seemed to have the highest secretion of these inflammatory cytokines compared to both fresh and day 28 samples (Figure 6 A–D). Secretion of angiopoietin-1, a protein that plays a role in vascular development, was significantly reduced in day 28 cells (Figure 6 E). Granulocyte colony-stimulating factor (GCSF), which stimulates the BM to produce cells, was increased in day 1 cryopreserved cells; however, this increase was not seen in the day 28 cells (Figure 6 F). Furthermore, intracellular adhesion molecule 1 (ICAM-1), important in cell-to-cell adhesion, was increased in cryopreserved cells, whereas tissue inhibitor of metalloproteinases 2 (TIMP-2), which plays a role in extracellular matrix degradation and suppression of EC proliferation [25], was reduced in the cryopreserved cells (Figure 6 G–H). The secretion of pro-vasculogenic cytokine basic fibroblast growth factor (bFGF), pro-myogenic cytokine insulin-like growth factor 1 (IGF-1) and chemotactic cytokines stromal cell derived factor 1 (SDF-1) and VEGF were all observed to be unaffected by cryopreservation (Figure 6 I–L).

### Cryopreservation does not Affect *in vivo* Angiogenic Potential of CACs

**Figure 7:**
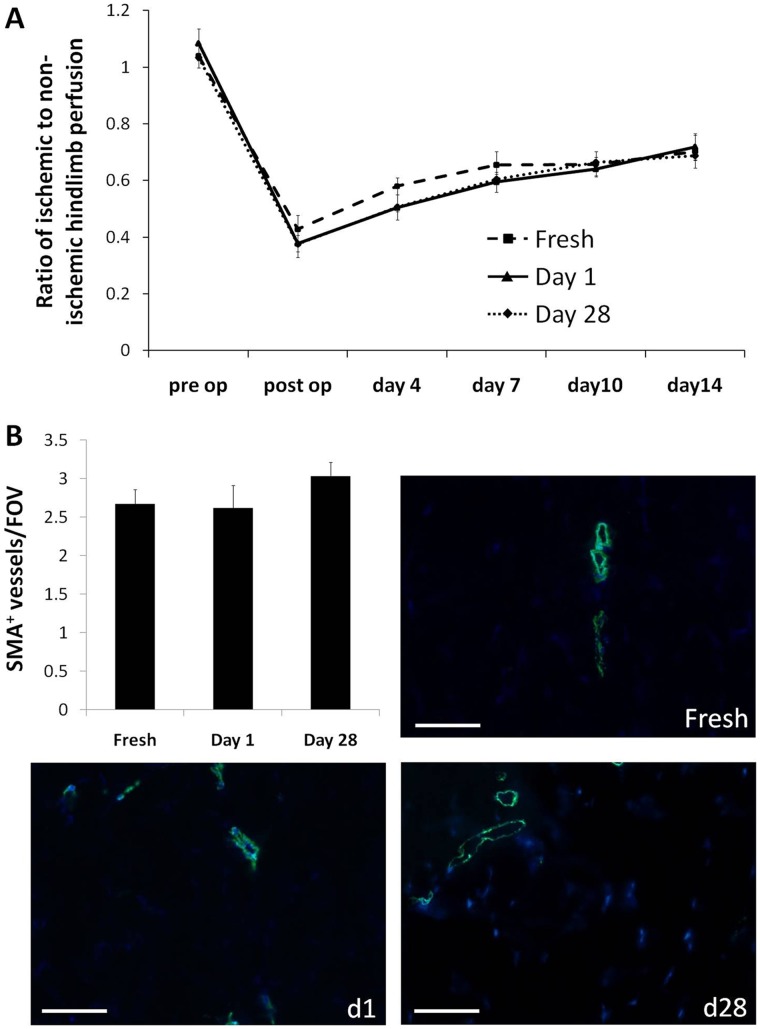
Cryopreservation does not affect the *in vivo* therapeutic function of CACs in a hindlimb ischemia model. Five million fresh, d1 or d28 cryopreserved-derived cells were injected into the skeletal muscle of mice that had undergone femoral artery ligation to induce hindlimb ischemia. Laser Doppler perfusion analysis was conducted over a period of 2 weeks (**A**). The tissue was harvested at 2 weeks and stained for SMA. The number of arterioles (per FOV ± SE) was determined (**B**), with representative images shown at 20× magnification (n = 5−6); scale bar = 50 µm.

Fresh or cryopreserved-derived human CACs were injected into the skeletal muscle of immunocompromised mice with hindlimb ischemia induced by femoral artery ligation. Laser Doppler perfusion analysis was conducted to measure blood flow over a 2-week post-treatment period, after which the animals were sacrificed. Animals receiving cryopreserved cell treatments displayed restoration in perfusion comparable to those receiving transplantation of fresh cells. No significant differences were observed in perfusion between treatments at days 4, 7, 10 and 14 post-op (Figure 7 A). Furthermore, there were no differences in the number of arterioles (assessed by smooth muscle actin (SMA) staining) between hindlimbs treated with cryopreserved cells or fresh cells (Figure 7 B). Staining the tissue with anti-human mitochondria antibodies yielded no detection of the transplanted human cells in the hindlimbs at the time of sacrifice, suggesting that transplanted cells did not persist in the tissue 2 weeks after delivery.

## Discussion

Cryopreservation is an appealing method for storing an individual’s own autologous stem and progenitor cells until needed for therapy; however, the effects of freezing cells for storage have not been fully characterized. In particular, there is limited data regarding cryopreserved PBMC-generated CACs, which have been a major focus of regenerative research. Our data demonstrates that cryopreservation does not adversely affect the viability of PBMCs and CACs but it does cause phenotypic changes in PBMCs and to a lesser degree in some subpopulations of CACs. However, the potent CD34^+^VEGFR2^+^CD133^+^ population remained unaffected in the CACs. Also the CACs demonstrated no changes in the secretion of important regenerative and chemotactic cytokines thought to be involved in the CAC’s regenerative ability via paracrine mechanisms. Furthermore, the angiogenic potential of CACs was examined *in vivo,* and no differences were seen between the 3 groups in their ability to restore perfusion to ischemic hindlimbs; demonstrating that cryopreservation did not affect the therapeutic potency of the cells. Nevertheless, some differences between the fresh and the cryopreserved CACs were observed, such as changes in inflammatory and other cytokine secretion and the numbers of cells expressing certain non-progenitor surface markers (L-selectin and CD31).

The maintenance of PBMC viability following cryopreservation observed in the present work is in accordance with other studies that also did not find considerable cell death after cryopreservation of PBMCs [26], [27]; however, it is believed that the cell viability achieved after cryopreservation is largely dependent on the expertise/technique of the laboratory that performs the cryopreservation [26], [27]. In particular, one report showed cell viability post-cryopreservation ranging from 1% to more than 90% [27].

The effect of cryopreservation on CAC viability has not been studied in great detail [28], [29], especially when considering PBMC-derived CACs. One investigation using 7-AAD, examined the viability of CACs from cryopreserved umbilical cord blood and found a significant level of apoptosis [29]. In contrast, our results illustrated no significant decline in CAC viability after 1 day and 28 days time points of cryopreservation. It is possible that this is attributable to differences between the cells under study or that after being cultured for 4 days the CAC viability differences are negated. The importance of cell viability after cryopreservation is imperative as CACs make up a small fraction of the total PBMCs. The ability to generate a large number of viable cells would allow for a better stem cell therapy outcome.

The cells’ phenotype was analyzed by staining for cell surface markers CD31, L-selectin, CD34, CD133 and VEGFR2 using flow cytometry. In our investigation, CACs generated from cryopreserved PBMCs showed no difference in expression of surface markers CD34, VEGFR2 and CD133; however a difference in CD31 and L-selectin expression was observed. CD34, CD133 and VEGFR2 are markers used to characterize CACs and the potent subpopulation of EPCs, although they are not exclusive to these populations and can be expressed by other hematopoietic lineages [30]. As previously mentioned, CD34^+^VEGFR2^+^CD133^+^ cells have been shown to be a potent subpopulation of cells involved in vascular regeneration [3]–[6]. The fact that this population and their vasculogenic derivatives (ex. CD34^+^VEGFR2^+^ cells) are unaffected by the cryopreservation in CACs, and are even increased in cryopreserved PBMCs, indicates that perhaps this population is able to better tolerate the harsh process of freezing without significant losses, compared to other populations.

CD31 is a marker of endothelial cells and L-selectin is an adhesion molecule found on CACs and other PBMCs. The expression of CD31 by day 28 cryopreserved PBMCs was increased; whereas the frequency of L-selectin^+^ PBMCs decreased, from about 85% to 70%, after 28 days of cryopreservation. This result is in accordance with previous studies that have also found down-regulation of L-selectin in PBMCs following cryopreservation [31], which may be a cellular response to stress [32]. Regarding progenitor markers CD34, CD133 and VEGFR2, we observed an increased expression following cryopreservation of PBMCs. Ketheesan *et al.* showed that the proportion of CD34^+^ cells in frozen cord blood did not change after freezing [33], while Lanza *et al*. demonstrated that 90-day cryopreserved PBMCs derived from patients with non-Hodgkin’s lymphoma had an increase in CD34^+^ cells compared to fresh samples, attributed to a decrease in mature myeloid cells [34]. Many hypotheses exist to explain the changes in phenotypes after cryopreservation: 1) more cells could indeed shift towards expression of particular markers during the cryopreservation process; 2) due to cell death of other subpopulations, there is a relative increase of more cells expressing markers CD34, CD133 and VEGFR2 used to identify EPCs; or 3) an increase in false positives as perceived by the flow cytometry analyses. Given that the observed non-specific binding IgG controls did not differ among time points, it is plausible that the observed changes are due to one of the first two hypotheses; however, it is not yet clear whether one or both are predominant reasons for changes in stored cell phenotypes.

Although studies have shown that L-selectin decreases in immunomagnetically-purified CD34^+^ CACs immediately after thawing, the cells do recover their expression of L-selectin after a short period of culture [35]. This may explain why, in our study, we observed a decrease in PBMC L-selectin expression, which were analyzed immediately, while the CACs were generated by a 4-day culture protocol before analysis, and demonstrated no change in L-selectin on day 1 cryopreserved cells and even an increased expression by day 28 cryopreserved cells. Surprisingly, the levels of CD31 were decreased in cryopreserved CACs in contrast to cryopreserved PBMCs where the expression of CD31 was unchanged or increased.

We also assessed the function of the populations’ capacity for lectin-binding and LDL uptake, which are further characteristics used to assess CAC populations [1], [2]. As expected, a higher percentage of cells in the CAC sample showed lectin binding and DiI-LDL uptake, compared to PBMCs. In PBMCs there was no significant difference between the number of fresh and cryopreserved cells staining positive for either one of these factors. However, for CACs a significant increase was seen for lectin binding and LDL uptake in 28 day cryopreserved cells. This is in contrast to an investigation by Mieno *et al.* that showed that LDL uptake and lectin-binding of EPCs is unaffected by cryopreservation [28].

The cells’ function was further assessed by their adhesion and VEGF-mediated migration capabilities. The adhesion of PBMCs was unaffected by cryopreservation, which is somewhat contradictory to the observed decrease in L-selectin after cryopreservation. Binding of L-selectin inside the cell results in the activation of β1 and β2 integrins which promotes adhesion of leukocytes to fibronectin [36]. It is possible that while the loss of L-selectin in PBMCs is statistically significant, it is not, however, physiologically relevant, as compensatory mechanisms may be in action (e.g. other selectins may assume similar functions [37]). The migration potential of PBMCs using VEGF for chemotaxis was also unaffected by cryopreservation. The fact that there was no change in VEGF-mediated migration even though there was increased expression of one of its receptors (VEGFR2) was not further investigated. One theory could be attributed to the newly expressed receptor proteins being non-functional, and therefore being unable to contribute to the cells’ migration.

CAC adhesion and migration capacities were mostly unaffected by cryopreservation. There is a significant decrease in CAC migration between fresh and 1-day frozen samples, but no significant difference between fresh and 28-day frozen samples. The loss of migratory function in the 1-day samples may be attributed to shock that the cells underwent during the short succession between freezing/thawing procedures; whereas the maintenance of migratory potential in the 28-day cells is in accordance with other studies showing that migration is not affected by cryopreservation [28], [38].

Secretion of cytokines is an important aspect of the CAC’s ability to repair tissue after an ischemic injury [10]. Using an array we looked at a variety of cytokines released by cultured fresh and cryopreserved CACs. Inflammatory cytokines were increased a few fold in the cryopreserved cells; and this increase was greatest in day 1 cryopreserved cells, even compared to day 28 cells. Why the day 1 cells increase their secretion of inflammatory cytokines remains unknown. As the cryopreservation preparation procedures are the same, the difference between day 1 and day 28 might be explained by the short period of time between the freezing/thawing procedures. While changes in inflammatory cytokine production in cryopreserved CACs have not been reported, PBMCs have been previously shown in some studies to have a different cytokine secretion pattern post-cryopreservation [39], [40]. In one study, 12 months frozen PBMCs were found to spontaneously show an increase of inflammatory cytokines IL-6, IL-10, IL-13 and IFN-γ [39]. In our study, we found that the secretion of several other cytokines was also changed after cryopreservation. ICAM-1, an adhesion factor and, in its soluble form, an indicator of endothelial dysfunction [41], was found to have an increased secretion in cells after cryopreservation. However, this might be attributed to the increase in IL-1α, IL-β and TNF-α which have been found to up-regulate ICAM-1 in ECs [42]. Similarly, the observed angiopoietin-1 and TIMP-2 decrease in our cryopreserved cells may also be attributed to the effect of the increased IL-1β and TNF-α secretion by the cells [43], [44]. While differences in the secretion of certain cytokines was observed in cryopreserved cells, some of the most important pro-regenerative and chemotactic cytokines such as IGF-1, bFGF, SDF-1α and VEGF remained unchanged between the fresh and cryopreserved CACs. Although some changes between fresh and cryopreserved cells were found, when their angiogenic potential was investigated *in vivo*, no differences in the recovery of perfusion or arteriole density were observed between the animals treated with cryopreserved cells vs. fresh cells, underscoring the conservation of therapeutic function after cryopreservation.

The extraction and cryopreservation of PBMCs is a well-established protocol, already in use for treatment of patients suffering with bone marrow cancers and other blood diseases. While the amount of serum used during our freezing step is 6% and other protocols have used storage solutions containing up to 90% serum [45], [46], we did not observe a great loss of viable cells. This is an important finding, considering that a study investigating cryopreservation of MNCs reported that viability of <70% is associated with compromised proliferative responses to antigens and mitogens, and are not suitable for cytokine production studies, flow cytometric analyses, or immunomagnetic cell separation [47], [48].

Overall, our data indicate that the function of therapeutic CAC populations is generally preserved after short- and longer-term cryopreservation, with the cells retaining their adhesion and migratory capabilities. Despite the more drastic phenotypic changes in PBMCs following cryopreservation, these cells can still generate CACs with no changes in the population of the potent vasculogenic CD34^+^VEGFR2^+^CD133^+^ cells compared to their fresh counterparts. Furthermore, the secretion of important regenerative and chemotactic cytokines is unchanged in the cryopreserved CACs along with their angiogenic potential *in vivo*. However, the cryopreserved CACs do show a significant increase in secretion of inflammatory factors, which may affect the expression of additional cytokines such as angiopoietin-1, GCSF, TIMP-2 and ICAM-1. Our findings also have clinical implication, as more research is showing that PBMCs and CACs have a positive impact on neovascularization in ischemic and cardiovascular diseases. One important factor for successful cell therapy is generating enough therapeutic cells to achieve sufficient repair of the damaged tissue. Progenitor cells from the peripheral blood are much easier to obtain than the traditional bone marrow harvest, which is more painful and requires anesthesia. The PBMCs may be collected over a given period and stored each time until enough cells are collected and the patient is primed to receive the cell therapy, at which time the PBMCs may be cultured to generate the therapeutic CACs.

### Conclusions

In conclusion, the CACs generated from the cryopreserved PBMCs show no significant difference in viability, in *in vitro* functions such as adhesion and migration, or in *in vivo* angiogenic potential; however, an increase in LDL uptake and lectin binding was observed. Also while there are some phenotypic changes, the therapeutically potent CD34^+^VEGFR2^+^CD133^+^ population remains unchanged. Additionally, the secretion of important regenerative and chemotactic cytokines involved in tissue repair through paracrine mechanisms is unaffected in CACs after cryopreservation. The ability to store these therapeutic cells for a long period of time without large cell losses and disruption of function will offer more patients an opportunity to experience successful cell therapy.

## Materials and Methods

A summary of the cell populations, methods and time points are presented in Figure 1.

### Ethics Statement

The study was approved by the Human Research Ethics Board of the University of Ottawa Heart Institute, and informed consent was obtained from all donors.

### Cell Isolation

Total PBMCs were isolated from 100 ml of fresh blood samples of seven healthy human donors both male and female (20–35 years of age) by Histopaque 1077 (Sigma-Aldrich, Oakville, Canada) density-gradient centrifugation as described previously [49]. The cells within the buffy coat were then either analyzed (baseline PBMCs), cultured to generate CACs, or cryopreserved.

### Cryopreservation

PBMCs retrieved from the buffy coat were reconstituted in a 1 ml solution of Isocove’s Modified Dulbecco’s Medium (IMDM; Stem Cell Technologies, Vancouver, Canada) containing 5% dimethyl sulfoxide (DMSO; Sigma-Aldrich) and 6% donor cell-matched serum and placed into a cryovial (Corning Incorporated, Corning, USA). The DMSO was mixed with the IMDM prior to addition of blood serum and cells in order to prevent clump formation. The cells were cooled to −80°C over 3 hours before being transferred to liquid nitrogen for storage.

For rapid thawing and to prevent toxicity to cells, cryovial tubes were taken from the freezer and immediately placed in a 37°C water bath until ice crystals disappeared, after which the cells were diluted 5× with IMDM and centrifuged at 1400 rpm and immediately resuspended in fresh Endothelial Basal Media (EBM-2; Clonetics, Guelph, Canada).

### CAC Culture

CACs were generated by seeding fresh PBMCs, or PBMCs thawed at days 1 or 28 post-freezing, on fibronectin (Sigma-Aldrich)-coated plates in EBM supplemented with EGM-2-MV-SingleQuots (Clonetics) containing 5% fetal bovine serum, human VEGF, human insulin-like growth factor 1, human epidermal growth factor and antibiotics for 4 days. After 4 days, the media was removed and the cells were lifted using gentle pipetting with PBS and prepared for subsequent assays.

### Flow Cytometry Staining

Cells were counted using a Vi-Cell analyzer (Beckman Coulter, Mississauga, Canada). PBMCs or CACs (3×10^5^) in 200 µl of EBM were stained for 30 minutes at 4°C with pre-conjugated antibodies against the following antigens: CD31-FITC (Beckman Coulter), CD34-PECy7 (BD Biosciences, Mississauga, Canada), KDR-PE (R&D Systems, Minneapolis, USA), CD133-APC (Miltenyl Biotec, Auburn, USA) and L-selectin-ECD (Beckman). Samples were also stained with appropriate IgG isotype-matched controls. Immediately prior to analysis, the viability stain 7-actinomycin D (7-AAD) (Invitrogen, Burlington, Canada) was added to samples to a final concentration of 8.3 µg/ml. Characterization also included incubating 3×10^5^ cells with 2 µg/ml of 1,1′-dioctadecyl-3,3,3′,3′-tetramethylindocarbocyanine–labeled acetylated low density lipoprotein (DiI-LDL) (Invitrogen) for one hour at 37°C after which the cells were pelleted, fixed for 10 minutes with fixation buffer (BD Biosciences) and further incubated for an hour at 37°C with 10 µg/ml of fluorescein isothiocyanate (FITC) labelled lectin from *Ulex europaeus* agglutinin-1 (Sigma-Aldrich). Cells were analyzed and quantified with a BD FACSaria cell sorter (BD Biosciences) until the number of events (cells) reached 200,000.

### Functional Assays

Cells were suspended in 50 µg/ml of 4′,6-diamidino-2-phenylindole (DAPI; Sigma-Aldrich) in PBS for 30 minutes at 37°C, after which the cells were pelleted and counted. **Migration.** DAPI-stained PBMCs or CACs (2×10^4^) were suspended in 100 µl of VEGF-free EBM and placed in the top compartment of a 24-well Boyden chamber (Corning Inc.) with the lower chamber coated with fibronectin and containing 350 µl of 0.05 µg/ml VEGF (Cedarlane, Burlington, Canada) in EBM. The cells were incubated for 24 hours in 5% CO_2_ after which they were fixed with 4% paraformaldehyde and washed with PBS. **Adhesion.** DAPI-stained cells (2×10^4^) were plated in 1 ml of EBM on fibronectin coated 24-well plates and incubated for 1 hour in 5% CO_2_ after which they were fixed with 4% paraformaldehyde. The number of DAPI stained cells for both assays were counted in 5 random fields-of-view for each well at 20× magnification using Olympus B×60 fluorescent microscope (Olympus Canada Inc., Markham, Canada). Assays were performed in duplicate.

### Cytokine Array

Three million PBMCs were plated on fibronectin-coated 6-well plates in 2 ml of EBM media. After culturing for 4 days to generate CACs, the media was collected. Cytokine arrays (RayBiotech, Norcross, USA) were incubated with 100 µl of media per sample according to manufacturer’s protocol. Following the cytokine array procedures, the membranes were exposed and the light produced at each spot (proportional to the amount of cytokine bound) was quantified using AlphaEaseFC.

#### Hindlimb ischemia mouse model

Nude BALB/C mice (7–8 weeks old) underwent ligation of proximal femoral arteries under 2% isoflurane, as previously described [14]. Subsequently, the ischemic hindlimb was injected downstream of the ligation site with 2 equivolumetric injections (50 µl total, suspended in PBS) containing a total of 5×10^6^ CACs that were derived from: i) fresh PBMCs; ii) day 1 cryopreserved PBMCs; or, iii) day 28 cryopreserved PBMCs, prepared as described above. Injections were performed with a 27 gauge needle. Prior to injection, cell viability was confirmed to be >85%.

### Laser Doppler

Hindlimb perfusion was measured using laser Doppler analysis pre-operatively, post-operatively, and at days 4, 7, 10 and 14, as described previously [14]. Briefly, while mice were anaesthetized with 2% isoflurane, single point measurements were recorded (moorLD12; Moor Instruments, Axminster, UK) in both hindlimbs and used to evaluate perfusion. The data is expressed as a ratio of ischemic to non-ischemic hindlimb blood flow.

### Immunohistochemistry

Two weeks post-CAC treatment, the mice were sacrificed and the ischemic hindlimb tissue was collected, frozen and sectioned. After fixing tissue sections with acetone for 10 minutes, the sections were washed for 3×5 minutes with PBS, blocked for an hour using 10% fetal bovine serum in PBS and stained with anti-SMA antibody (1∶100; abcam, Cambridge, USA). Following 3 washes (5 minutes each) and secondary antibody application (1∶600 anti-rabbit Alexa 488; Invitrogen,) the slides were mounted with DAPI and analyzed at 20× magnification with an Olympus BX50 microscope using Image Pro Plus. The number of arterioles was counted by 2 blind observers and expressed as the average number of SMA^+^ vessels per field of view (FOV). Additionally, to visualize the injected CACs, hindlimb sections were stained with anti-human mitochondria antibody (1∶100; Chemicon, Temecula, USA), following the manufacturers instruction.

### Statistical Analysis

Results obtained for each phenotype and each cell type were analyzed for differences between days using a one-way Anova adjusted for donor. Sub-analyses were performed when a Bonferonni significant result was obtained using a paired t-test. Results were Bonferonni adjusted for the number of tests performed for each of viability, phenotype and function separately, to achieve an alpha of 0.05; *p*-values given for sub-analyses are not corrected. Statistical analyses were performed using R (R development core team, Vienna, Austria).
